# Image-Based Computational Hemodynamics Analysis of Systolic Obstruction in Hypertrophic Cardiomyopathy

**DOI:** 10.3389/fphys.2021.787082

**Published:** 2022-01-06

**Authors:** Ivan Fumagalli, Piermario Vitullo, Christian Vergara, Marco Fedele, Antonio F. Corno, Sonia Ippolito, Roberto Scrofani, Alfio Quarteroni

**Affiliations:** ^1^MOX, Dipartimento di Matematica, Politecnico di Milano, Milan, Italy; ^2^LaBS, Dipartimento di Chimica, Materiali e Ingegneria Chimica “Giulio Natta”, Politecnico di Milano, Milan, Italy; ^3^Children’s Heart Institute, Hermann Children’s Hospital, McGovern Medical School, University of Texas Health, Houston, TX, United States; ^4^Radiology Unit, L. Sacco Hospital, Milan, Italy; ^5^Cardiac Surgery Unit, L. Sacco Hospital, Milan, Italy; ^6^Institute of Mathematics, École Polytechnique Fédérale de Lausanne, Lausanne, Switzerland

**Keywords:** hypertrophic cardiomyopathy, septal myectomy, cardiac cine-MRI, image-based computational fluid dynamics, mitral valve, patient-specific simulations

## Abstract

Hypertrophic Cardiomyopathy (HCM) is a pathological condition characterized by an abnormal thickening of the myocardium. When affecting the medio-basal portion of the septum, it is named Hypertrophic Obstructive Cardiomyopathy (HOCM) because it induces a flow obstruction in the left ventricular outflow tract. In any type of HCM, the myocardial function can become compromised, possibly resulting in cardiac death. In this study, we investigated with computational analysis the hemodynamics of patients with different types of HCM. The aim was quantifying the effects of this pathology on the intraventricular blood flow and pressure gradients, and providing information potentially useful to guide the indication and the modality of the surgical treatment (septal myectomy). We employed an image-based computational approach, integrating fluid dynamics simulations with geometric and functional data, reconstructed from standard cardiac cine-MRI acquisitions. We showed that with our approach we can better understand the patho-physiological behavior of intraventricular blood flow dynamics due to the abnormal morphological and functional aspect of the left ventricle. The main results of our investigation are: (a) a detailed patient-specific analysis of the blood velocity, pressure and stress distribution associated to HCM; (b) a computation-based classification of patients affected by HCM that can complement the current clinical guidelines for the diagnosis and treatment of HOCM.

## Introduction

Hypertrophic Cardiomyopathy (HCM) is a typically congenital cardiac disorder characterized by an abnormal thickening of the myocardium (wall thickness exceeding 15 mm, in adult patients) without additional causes inducing secondary hypertrophy (Elliott et al., 2014). The prevalence is 0.2–0.6% in the Western world population, with a reported annual mortality rate of 1%. When the thickening affects the medio-basal region of the interventricular septum, this condition may cause a flow obstruction in the Left Ventricular Outflow Tract (LVOT), and thus it is named Hypertrophic Obstructive Cardiomyopathy (HOCM). People with mild forms of HOCM often remain oligosymptomatic or even asymptomatic for many years. Otherwise, they may develop dyspnea, angina pectoris, or stress-induced syncope, with an increased risk of sudden cardiac death, particularly in young people and athletes, more easily exposed to physical efforts that require a sudden increase of the cardiac output. The pathological effects of HOCM can be worsened by concurrent conditions increasing the ventricular pressure afterload, such as systemic hypertension and/or aortic valve stenosis. The LVOT obstruction is dynamical, and largely influenced by changes in left ventricular pressure overload and contractility, with subsequently increased systolic pressure in the Left Ventricle (LV) and possible secondary Mitral Valve Regurgitation (MVR), myocardial ischemia, and reduction in cardiac output. In particularly severe conditions, a Systolic Anterior Motion (SAM) of the mitral valve can occur, thus further worsening the LVOT obstruction (Jiang et al., 1987; Geske et al., 2012; Ibrahim et al., 2012; Sherrid et al., 2016; Akiyama et al., 2017).

One of the most frequently employed surgical treatments for a pathologically relevant HOCM is the septal myectomy, namely the resection of a portion of the interventricular septum, to abolish or reduce the obstruction in the LVOT (Morrow et al., 1975; Ommen et al., 2005; Elliott et al., 2014; Maron and Nishimura, 2014; Nicolò et al., 2019). Therefore, identifying the location and extension of the septal region responsible for such obstruction is of paramount importance to guide the preoperative design of the surgical procedure.

To obtain quantitative indications on velocity patterns, pressure gradients and wall shear stresses associated to the ventricular blood flow dynamics, computational hemodynamics approaches have proved to be extremely helpful, thanks to their flexibility and level of detail (Quarteroni et al., 2017). In this regard, two main standpoints are currently adopted: Fluid-Structure Interaction (FSI) simulations and prescribed-motion Computational Fluid Dynamics (CFD). The first approach consists in looking for the coupled solution of the fluid dynamics of blood flow and of the structure mechanics of the myocardium and cardiac valves, thus requiring a proper calibration of the mechanical parameters of the tissue, and possibly entailing a very high computational cost (Kunzelman et al., 2007; Su et al., 2014; Gao et al., 2017; Lassila et al., 2017; Karabelas et al., 2018; Collia et al., 2019; Feng et al., 2019; Kaiser et al., 2020; Meschini et al., 2021). On the other hand, in image-based CFD, the patient-specific displacement of the myocardium and valves leaflets is reconstructed from kinetic medical images (such as cardiac cine-MRI) and then prescribed as endocardial displacement to obtain the fluid domain configuration. This latter approach, by reducing the mathematical complexity of the problem with respect to a full FSI system, at the expense of requiring a more complex image processing procedure, has provided insightful indications on cardiovascular diseases in a series of reports (D’Elia et al., 2011; Seo et al., 2014; Chnafa et al., 2016; Otani et al., 2016; Su et al., 2016; Bavo et al., 2017; This et al., 2020), including the computational study of HCM (Deng et al., 2018; Nardi et al., 2019; Fumagalli et al., 2020). In all the quoted references, the reconstruction of the endocardium geometry was mainly based on the sole short-axis acquisition series, whereas long-axis views are only employed to identify some specific points or distances. This limitation was mainly due to the relative difficulty of combining the data from different acquisitions.

The goal of this study was to investigate the hemodynamics in the systolic phase and the LVOT obstruction severity and extent in patients with HCM, and also to provide quantitative information useful to potentially guide the design of the possible surgical treatment. To these aims, we used a computational procedure based on cardiac cine-MRI data (Fumagalli et al., 2020), and we introduced further improvements for the integration of short-axis and long-axis views. By the reconstruction procedure that we propose and employ in the present study, we merge all the cine-MRI series in a single image: this entails the extraction of all the geometric information from the imaging data, thus yielding an improved level of detail in the reconstructed geometry and displacement of the LV with respect to the standard reconstructions based on the sole short-axis acquisition series. The ventricular motion was employed to set up the boundary conditions at the wall for the CFD analysis, and it was then extended to the mitral valve leaflets, whose effects on the hemodynamics were accounted for by a resistive method (Fedele et al., 2017; Fumagalli et al., 2020).

The novelty of this study consists in the quantitative comparison of patients with some types of HCM by means of the extended computational procedure outlined above, based on routinely acquired cardiac cine-MRI data. This results in a CFD-based assessment and classification of the severity of the HCM-induced flow obstruction with *ad hoc* designed hemodynamical indicators, and the identification of the region of the septal wall most suitable for the surgical approach by septal myectomy. The systematic discussion of the outcomes represents a step forward toward the applicability of our computational tools to accompany standard diagnostical procedures, and the definition of a comprehensive severity score for HOCM including hemodynamics indicators.

## Materials and Methods

In the present section, we describe the imaging data on which this study is based, and we introduce a computational procedure encompassing image processing, surface morphing, and numerical simulations for the study of the hemodynamics in LV and ascending aorta.

### Patients Data

Cardiac cine-MRI data of three patients were provided by L. Sacco Hospital in Milano, Italy, with the approval of the Ethics Committee and in accordance with the ethics guideline of the institutions involved, including the signed consent of the patient. The main characteristics of the patients are reported in Table 1.

**TABLE 1:** Main characteristics of the HCM patients investigated.

Patient	1	2	3
Age	57	66	83
Sex	F	M	F
EDWM [g]	240	216	98
Max IV septum thickness [mm]	25	18	16
Right ventricle free wall	hypertrophic	regular	regular
MV regurgitation	NO	NO	NO
Description of LV hypertrophy	concentric, symmetric HCM, and amyloidosis infiltrations	concentric HCM, distributed hypokinesis	symmetric HCM, preserved global systolic function

*EDWM is the End-Diastolic Wall Mass, that is the mass of the LV myocardium.*

The following are the clinical features of the patients:

• Patient 1. The overall contractility of the right ventricle is preserved, despite the hypertrophy involving also the free right ventricular wall, and in contrast with the reduced overall systolic function of the LV. She also presented with a mild pericardial effusion. No valve regurgitation or stenosis were reported.
• Patient 2. This patient is a smoker, hypertensive and overweight; he experienced chest pain and incipient dyspnea, and was recently hospitalized for heart failure. Subsequent electrocardiography limited the electrophysiological findings to sporadic extrasystoles. A trivial degree of regurgitation was reported for the aortic and tricuspid valves, but not for the mitral valve. The right ventricle had a significantly reduced systolic function (ejection fraction = 12%) although the values of its walls thickness remained in the normal range.
• Patient 3. No effusions, edemas, fibrosis, or other concurrent conditions were reported, except for a prolapse of the anterior leaflet of the mitral valve toward the septum (SAM), without regurgitation.

For each patient, the data included different views, with different resolution properties: a volumetric short-axis acquisition, with a spacing and a slice thickness of 8 mm along the LV main axis, a space resolution of 1 mm and a time resolution of 1/20 of the heartbeat; a set of single-slice, two-dimensional long-axis acquisitions on the so-called two-chambers, three-chambers, and four-chambers planes, with space resolution of 1 mm and time resolution of 1/20 or 1/30 of the heartbeat. These are standard cardiac cine-MRI data, routinely acquired during diagnostical procedures: the reconstruction algorithm presented in this study does not require the setup of *ad hoc* acquisitions.

So far, none of these patients underwent surgery yet, based on the indications given by their attending clinicians.

### Reconstruction of Geometry and Motion

**FIGURE 1:**
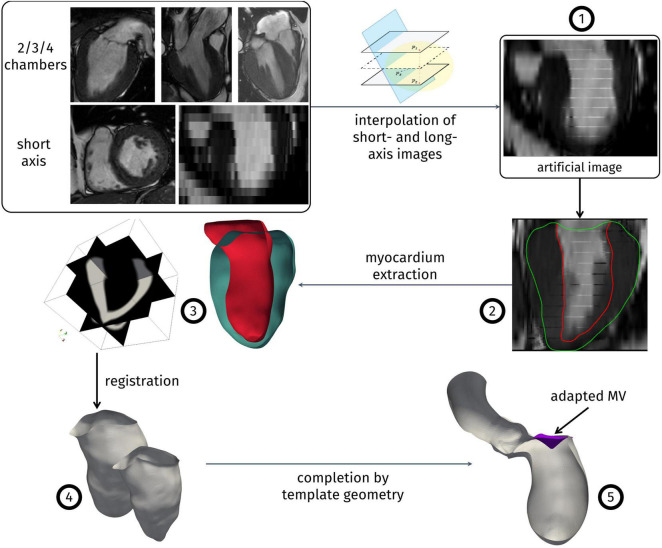
Reconstruction algorithm. Numbers correspond to the steps of Algorithm 1.

Since the only available three-dimensional data was represented by the short-axis views, which, however, has a relatively low resolution along the LV main axis, we developed an algorithm to enhance the short-axis images with the long-axis acquisitions (commonly named as 2/3/4-chambers views) obtaining a high-resolution artificial, time-dependent series of volumetric image. This represents the first step of our reconstruction algorithm, described in the following Algorithm 1 and depicted in Figure 1.

**Algorithm 1 – Reconstruction procedure.**

1. Create a high-resolution artificial image merging the information from all the available cine-MRI series. This operation encompasses:
1.a. creating an empty artificial image initialized with the short-axis bounding box and with a uniform space resolution of 1 mm in the three cartesian directions;
1.b. for each target pixel of this new artificial image, identify the nearest pixel on each slice of the input cine-MRI series;
1.c. compute the new gray level as a weighted average of the values of the pixels identified at point b, with the weights depending on their distance from the target pixel;
2. for each instant of the artificial image, segment both the endocardium and the epicardium of the LV;
3. intersecting the endocardium and epicardium surfaces obtained at the previous step, obtain a volumetric representation of the myocardium as a level-set image;
4. choosing the end of systole as a reference configuration, apply a registration algorithm among the level-set images of the myocardium, to obtain a displacement field for each acquisition time. The displacement field is applied to the endocardium surface of the reference configuration to obtain its evolution throughout the heartbeat;
5. complete the geometry resorting to a template geometry: from the Zygote solid 3D heart model (Zygote Media Group Inc., 2014), a healthy heart complete geometry reconstructed from CT-scans, we extract the aorta and the mitral valve leaflets. The templates are adapted to the patient-specific LV, based on its aortic and mitral valves annulus, to obtain a complete computational domain for the study of the systolic phase, and the displacement field obtained in step 4 is extended to the whole geometry.

The reconstruction procedure presented above is based on the one we previously reported (Fumagalli et al., 2020). The main advancement is represented by the generation of the enriched artificial images described at step 1 and their employment to accurately capture the patient-specific aorto-mitral annulus (exploited at steps 3 and 5) and the shortening and elongation of the LV directly from segmentation, without the need to measure it separately. The merging tools presented in the first step of the algorithm have been implemented in MATLAB (SCR_001622). As reconstruction tools, we employed the Medical Image Toolkit (MITK)^1^ (Wolf et al., 2005; Nolden et al., 2013) for the segmentation step 2, a procedure based on SimpleITK^2^ for the registration step 4, whereas for the other steps and for the automatic generation of the hexahedral mesh we employed the tools presented in Fedele and Quarteroni (2021) and other *ad hoc* semi-automatic tools hinging upon the Visualization Toolkit (VTK, SCR_015013)^3^ and the Vascular Modeling Toolkit (VMTK, SCR_001893)^4^ (Antiga et al., 2008).

Regarding the mitral valve, the use of a template geometry was motivated by the fact that, usually, standard MRI acquisitions do not provide sufficient details to allow an accurate reconstruction of the valves leaflets. Since we only investigated the systolic phase, and since mitral regurgitation was not present in any of the patients, the valve was maintained closed during the whole systolic phase. However, we believe it was important to include the mitral valve in the domain, since it defines two thirds of the LVOT boundaries and thus influences the blood dynamics. For all the patients we considered the same mitral valve geometry template, adapted to the patient’s annulus as described in point 5 of Algorithm 1. In particular, for Patient 3 we do not consider the SAM of the valve, in order to isolate and analyze only the HCM-induced effects on the blood flow.

### Mathematical Model – Computational Fluid Dynamics

**FIGURE 2:**
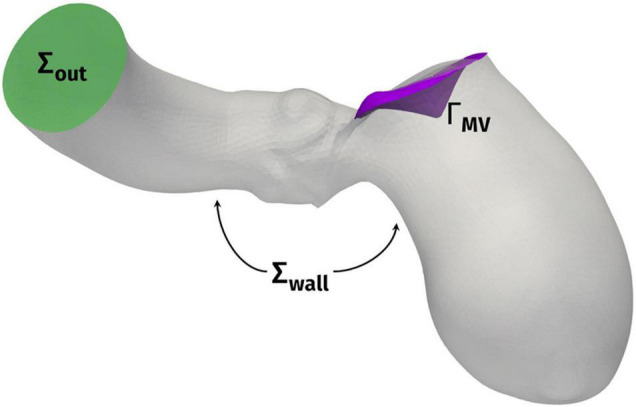
Computational domain Ω with its outlet section Σ_out_ and the endocardial and aortic walls Σ_wall_. Γ_MV_ denotes the mitral valve leaflets.

The computational domain, comprising the LV, the ascending aorta, and the surface Γ_MV_ representing the mitral valve leaflets, is displayed in Figure 2. We denote by Σ_out_ the outlet section of the ascending aorta and by Σ_wall_ the boundary comprising the ventricular endocardium and the aortic wall. Under the common assumption that blood is an incompressible Newtonian fluid, at least in the heart chambers and large vessels, we could model its flow by incompressible Navier-Stokes equations, with density ρ = 1.06 × 10^3^ kg/m^3^ and viscosity μ = 3.5 × 10^–3^ Pa × s (Quarteroni et al., 2019). The ventricle contraction and the displacement of the domain, reconstructed from the imaging data as described in the previous section, is prescribed as wall motion for the fluid problem and incorporated in the model by an Arbitrary Lagrangian-Eulerian (ALE) formulation of Navier–Stokes equations (Donea et al., 1982; Formaggia and Nobile, 1999) whereas the mitral valve Γ_MV_ is modeled as a surface immersed in the fluid dynamics domain by the Resistive Immersed Implicit Surface (RIIS) method. RIIS lays in the class of immersed boundary methods (Peskin, 1972; De Hart et al., 2003; Iaccarino and Verzicco, 2003; Mittal and Iaccarino, 2005; van Loon et al., 2006; Griffith et al., 2009; Borazjani et al., 2010; Griffith, 2012; Borazjani, 2013; Kamensky et al., 2015; Wu et al., 2018). It was previously introduced by us (Fedele et al., 2017), based on the Resistive Immersed Surface method developed by another group (Fernández et al., 2008; Astorino et al., 2012), and it has been already applied by us in the study of the Systolic Anterior Motion (SAM) of the mitral valve (Fumagalli et al., 2020). A comprehensive discussion on the numerical modeling of heart valves is available in the literature (Yoganathan et al., 2005; Sotiropoulos and Borazjani, 2009; Votta et al., 2012; Marom, 2015; Mittal et al., 2016; Quarteroni et al., 2019). Since we focused our study on the systolic phase, and we want to analyze the effects of only the HCM-associated ventricular shape and motion on the blood flow, we prescribed a physiological pressure waveform, derived from Wiggers diagrams (Wiggers, 1923), as outflow condition at the distal section Σ_out_ of the ascending aorta. Because of the same reason, and as the main focus of the study was the assessment of the intraventricular obstruction, we did not include the aortic valve in the computational model.

**FIGURE 3:**
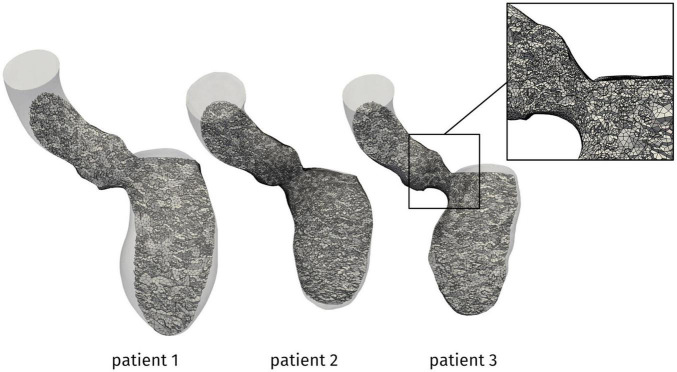
Computational mesh for the patients under investigation. In the box, a zoom showing the local refinement in the LVOT.

The problem was numerically solved by means of a first order semi-implicit time discretization and a SUPG/PSPG-stabilized piecewise linear Finite Element Method for space discretization of both velocity and pressure [see (Tezduyar and Sathe, 2003; Bazilevs et al., 2007; Forti and Dedè, 2015; Fumagalli et al., 2020) for further details], implemented in life^×5^, a multiphysics high-performance library based on the deal.II core^6^ and developed in the iHEART project.^7^ For all the patients, we discretized the domain by a hexahedral computational mesh with an average size of *h* = 1 mm and a local refinement to *h* = 0.3 mm in the region of the mitral valve and the LVOT, as shown in Figure 3. A simulation timestep of Δ*t* = 10^–4^ s is adopted, and a smooth spline interpolation was used to represent the reconstructed displacements on the simulations time grid. We performed a mesh convergence test ensuring that no significant differences may be found by using a finer mesh or a smaller timestep.

### Outputs of Interest

The reconstruction procedure and the computational methods described above allowed to obtain different relevant outputs about the overall cardiac function and the hemodynamics of the patients under investigation. In addition to well-known biomarkers such as the stroke volume (SV), the end-diastolic/end-systolic volume (EDV/ESV) and ejection fraction (EF), an accurate reconstruction of the LV volume changes occurring throughout the heartbeat was recovered. Then, focusing on the systolic phase, we could assess the possible HCM-induced obstruction in terms of velocity distribution, aortic jet development and pressure gradients. A particularly relevant indicator for the clinical assessment of the obstruction severity is the distribution of pressure along the septum. This information provides a quantitative measure of the spatial location and extension of the obstruction, very useful for the design of the surgical approach for HOCM: septal myectomy. Finally, thanks to the additional functionality offered by CFD with respect to diagnostical tests, we analyzed the Wall Shear Stress (WSS) on the endocardium and the aortic wall, which affects wall cell growth and possible flow-induced damage (Dolan et al., 2013), and evaluated the turbulent and vortex structures developing in the aorta by the Q-criterion (Hunt et al., 1988).

## Results

In this section, we present the outcomes of the proposed computational procedure for the data of three patients described above, in terms of geometric, functional, and hemodynamics indicators.

**FIGURE 4:**
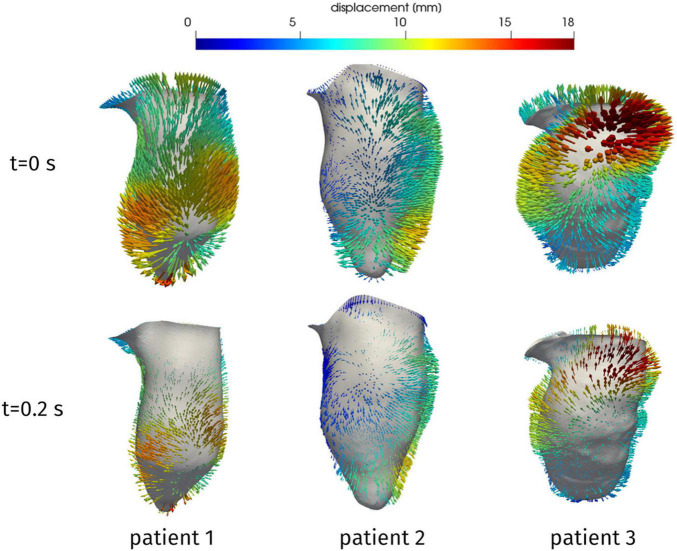
Reconstructed displacement field of the three patients, at the end of diastole (*t* = 0 s) and in late systole (*t* = 0.2 s). LV aligned vertically, with septal wall on the left.

**FIGURE 5:**
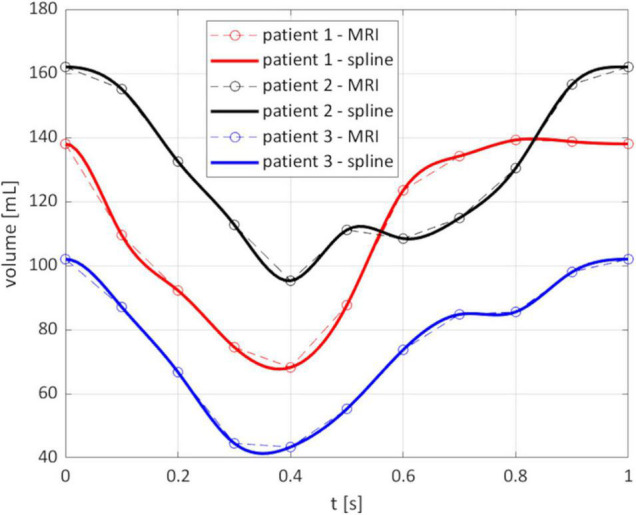
Evolution of the reconstructed volume of the LV cavity. Circled points: instants of the MRI segmentation; solid line: spline interpolation on all the times of the simulation.

From the reconstruction procedure we obtained the displacement fields shown in Figure 4. The distribution of displacement and its intensity are significantly different among the three patients under investigation, expressing the high variability of the effects of HCM on the ventricular contractility. Indeed, we observe a generally reduced motion of the septal wall, and at the same time we can notice that for Patient 1 this reduction is mostly concentrated in the region near to the ventricular base, whereas Patient 2 has a more homogenously distributed hypokinesis. These differences in the movement of the endocardial wall are reflected in the evolution of the volume of the LV cavity (Figure 5). A remarkable difference in this sense is displayed by Patient 2, with a slowed diastolic expansion after the end of systole, at 0.4 s. This is in accordance with the reduced ejection fraction of the patient (Table 2). In the same table, we can also compare the volume measurements obtained by our reconstruction with those estimated during the data acquisition. The end diastolic volumes (EDV) show a relatively good agreement between the reconstructed and estimated values, with a relative difference of less than 8% for all patients. On the other hand, the discrepancies between the two sources of data can be explained by the fact that the clinical estimates of volumes from cardiac cine-MRI are based on the approximation of LV as an ellipsoid (Hergan et al., 2008), which may not be particularly accurate for the end systolic volume (ESV), especially in the case of patients with HCM, for which contraction may be spatially inhomogeneous, and thus the geometry of the ventricle can present significant distortions.

**TABLE 2:** End-diastolic volume (EDV), end-systolic volume (ESV), and ejection fraction (EF) as reconstructed from the computational procedure (above) and from clinical estimations (below).

PT	EDV [ml]	ESV [ml]	EF [%]
1	138	68	51
2	162	94	42
3	102	43	57

1	128	81	37
2	152	103	32
3	101	37	63

**FIGURE 6:**
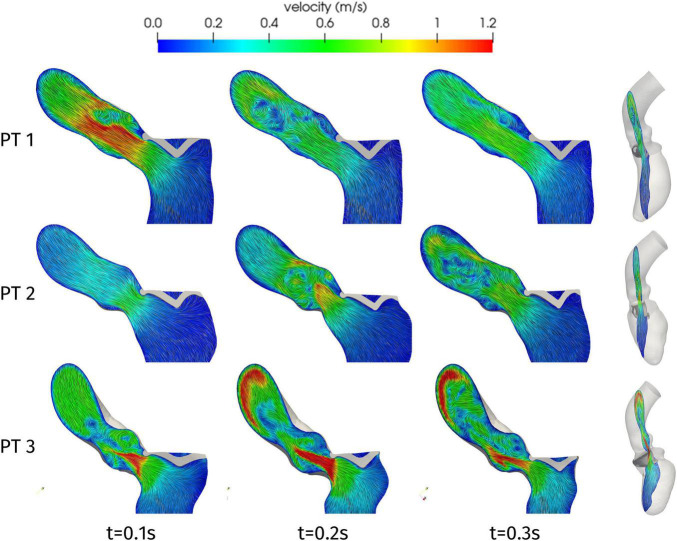
Velocity distributions on a two-dimensional slice of the LV and ascending aorta, orthogonal to the latero-lateral axis of the LV (as shown on the right), at significant times during systole.

Having discussed the reconstruction of the ventricular displacement, we now present the results of the computational hemodynamics simulations under such prescribed motion. As the aim of the present study was to assess the possible obstructions induced on the flow by HCM, we focus on the systolic phase. Figure 6 shows the blood velocity field on a longitudinal slice for the three patients at three different times during the systolic phase. As a first aspect, we can notice significant differences in the timing of the systolic peak. Patient 1 shows a very quick blood acceleration in the early stages of the ejection, with a strong jet involving the whole aortic root and a maximum velocity of 1.38 m/s attained at *t* = 0.08 s, then followed by a relatively slower deceleration. Patient 2 and 3, instead, present a more progressive development of the aortic jet, with a maximum velocity of 1.31 and 2.2 m/s at times *t* = 0.23 and 0.20 s, respectively, and persistent high velocity values (>1 m/s) also at later stages of the systolic phase. These two patients also have in common the impingement of the jet on the wall of the aorta just downstream to the Valsalva sinuses (superior wall for Patient 2 and inferior wall for Patient 3), although with different velocity values. In terms of velocity peak, Patients 1 and 2 lay just above the limits of the range of physiological values (1–1.2 m/s), whereas Patient 3 attains a pathological value.

**FIGURE 7:**
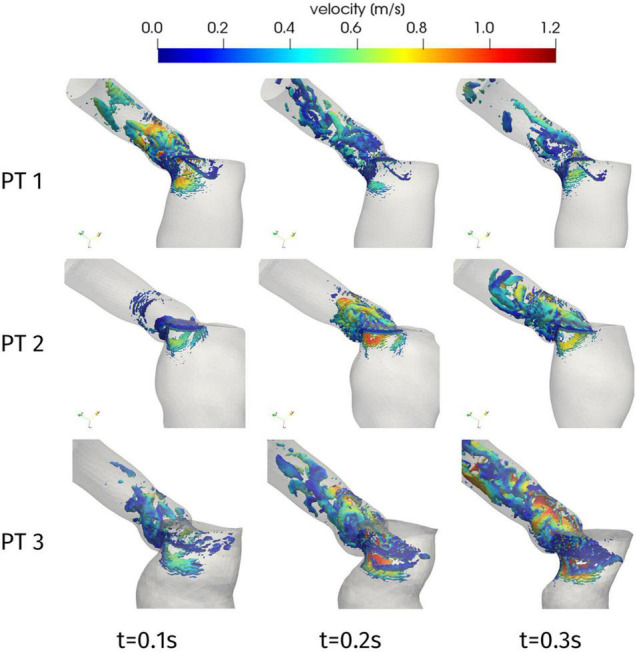
Q-criterion contours [10 log-spaced values for Q in 2.4 × (10^4^, 10^6^) s^– 1^] colored by velocity magnitude at significant times during systole.

Further information on the blood flow can be obtained from the Q-criterion representation of Figure 7, which identifies vortex structures in the flow (Hunt et al., 1988). In the case of Patient 1, we can notice that the aortic jet develops in the early systole, due to the systolic peak occurring at *t* = 0.08 s, and the corresponding large coherent structure visible in Figure 7 at *t* = 0.1 s rapidly splits up into smaller structures that are progressively breaking down along the flow. A similar behavior is shown by Patient 2 (apart from the systolic peak occurring later in systole, at *t* = 0.23 s), with the breakdown of the jet into disorganized structures being mainly localized in the aortic root. This is consistent with experimental and computational studies that can be found in the literature, e.g., (Sotiropoulos et al., 2016; Becsek et al., 2020), where the vortex-to-vortex interaction is shown to yield a transition to a flow state dominated by small eddies in a short time and near the distal section of the aortic root: our computational model is able to reproduce such main features, which are associated to a flow in transition to turbulence, despite the absence of the aortic valve leaflets which would introduce more accurate results at the expense of a more complex pre-processing procedure. Regarding Patient 3, the higher peak velocity, that lays in the pathological range, entails the generation of larger vortex structures as well as their persistence in the whole ascending aorta: a distinct transition to the turbulent regime characterizes this case (maximum Reynolds number in the aortic root: Re = 12000).

**FIGURE 8:**
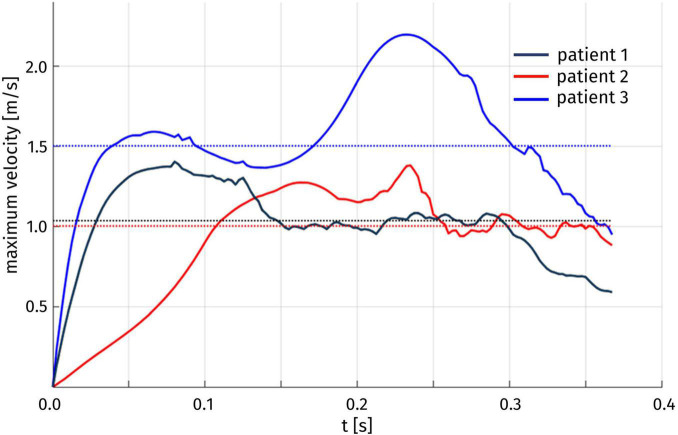
Time evolution of the maximum blood velocity U(t) over the whole aorta. The median U_thr_ of U(t) over time for each patient is indicated by a dotted line.

The velocity field can also be inspected to assess the *duration* of the HCM-induced obstruction. In particular, we introduce the quantity U(t) corresponding, for each time t, to the maximum blood velocity attained in the aorta, and we report its time evolution in Figure 8. For each of the three patients, we can identify a time interval in which significantly higher velocity values are attained, with respect to the rest of the systole. To provide a quantitative definition of this interval, we denote by U_thr_ the median value of U(t) over the whole systolic phase; then, we can define the duration of the obstruction as the length of the largest time interval in which the maximum velocity remains above U_thr_. The results are reported in Table 3. For the three patients, we can notice that the systolic obstruction occurs during about the 30% of the systole. This evaluation is particularly significant for Patient 3, which is the only one in which pathologically high peak velocity values are attained, as mentioned above. Similar considerations can be drawn from the evolution of the difference between the average pressure in the LV and in the Valsalva sinuses: a detailed analysis of the pressure gradient distribution is presented in the following.

**TABLE 3:** Duration of the obstruction and threshold value for its identification.

PT	Obstruction duration [s]	Threshold value U_thr_ [m/s]
1	0.12	1.04
2	0.15	1.00
3	0.13	1.50

**FIGURE 9:**
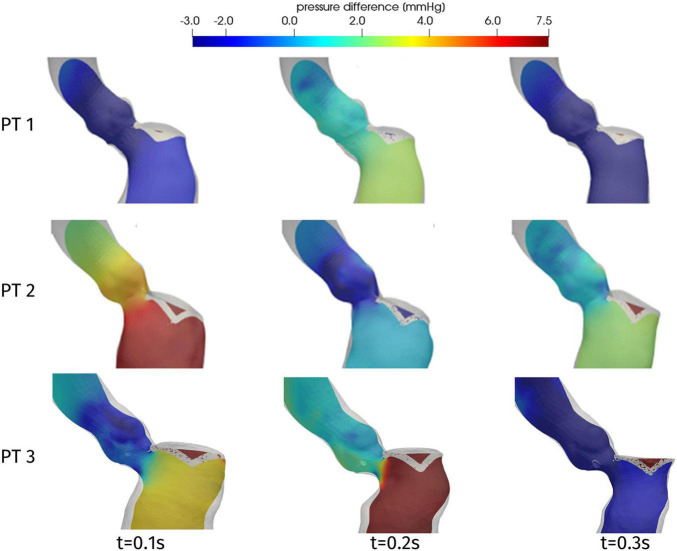
Distribution of pressure difference Δp = p-p_out_ on a longitudinal slice, at significant times during systole.

One of the main quantities that is inspected in the assessment of the hypertrophy-induced obstruction is the intraventricular pressure gradient, that is the variation of pressure in the LVOT. Figure 9 displays the distribution of the difference Δp = p-p_out_ between the pressure p and its value p_out_ at the outlet section Σ_out_ of the ascending aorta. For all patients and at all times, significant pressure gradients can be appreciated as expected in the LVOT and (at a smaller extent) in the Valsalva sinuses, whereas pressure is essentially uniform elsewhere. The overall pressure gradient of Patient 1 is always between −3 and 3 mmHg, whereas for the other two cases it is more than double. Regarding time evolution, each patient experiences an increase or a decrease of Δp at different stages of the systole, reflecting the variability that we observed above about the time dependence of the displacements and volume of the LV.

**FIGURE 10:**
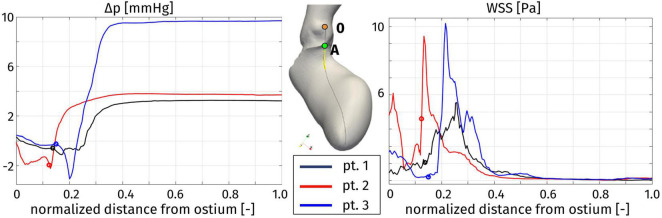
Distribution of pressure difference with respect to the Valsalva sinuses (left) and wall shear stress (right) along a line on the septum (center), at the instant of maximum pressure gradient: Patient 1, *t* = 0.08 s; Patient 2, *t* = 0.16 s; and Patient 3, *t* = 0.20 s. The distance is normalized with respect to the total length of the line. For each patient, the origin of the line in the right coronary ostium is denoted by 0, while the position of the aortic annulus (point A in the center figure) is indicated by a circle.

In order to provide a synthetic evaluation of the severity of the hypertrophy-induced obstruction, and to quantify its localization and extension, Figure 10 displays a plot of the pressure difference Δp on a line running along the septum. For each patient, the line we consider is identified as the intersection between the domain boundary and the plane passing through the right coronary ostium, the LV apex, and the center of the interventricular septum (see Figure 10, center). The pressure difference Δp is plotted against a normalized (with respect to the total length of the line from the ostium to the LV apex) coordinate running along the line, starting from the ostium. Each of these curves refers to the time with the recording of the highest pressure gradient, for every patient. As a first comment, this plot confirms that the whole pressure gradient essentially develops in the LVOT and the aortic valve orifice. In particular, for each patient, the spatial position of the flow obstruction is identified by the sudden increase in pressure, that is notably significant for Patient 3, for which the interventricular gradient max(Δp)-min(Δp) = 12 mmHg is definitely larger than the typical physiological pressure difference between LV and ascending aorta (<5 mmHg, [Elliott et al., 2014]). In the case of Patient 3 we also observe a pressure drop concentrated in the subaortic portion of the LVOT.

**FIGURE 11:**
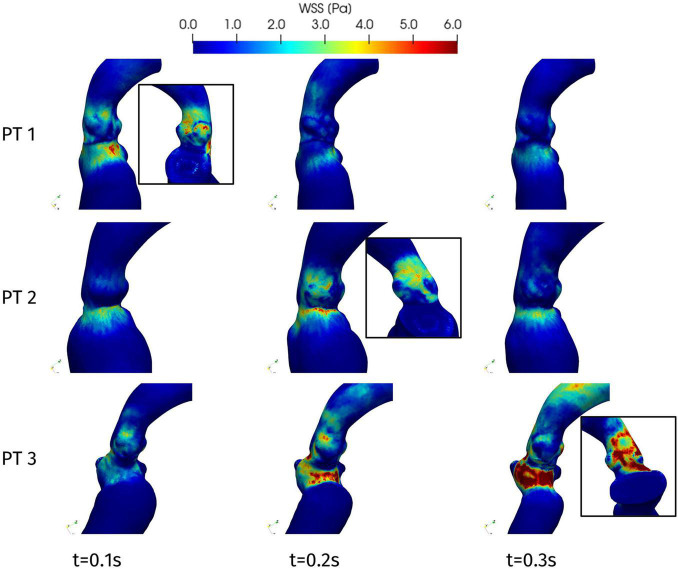
Wall shear stress distributions at significant times during systole: view from the interventricular septum. In the boxes, WSS distribution on the aortic root wall at the systolic peak (view from the atrial side): Patient 1, *t* = 0.08 s; Patient 2, *t* = 0.23 s; and Patient 3, *t* = 0.20 s.

Figure 11 represents the WSS distribution on the endocardium and aortic wall. The higher values of WSS are attained at the aortic root, particularly at the systolic peak, and at the septal wall of the LVOT, for all patients: in the first tract of the aorta these stresses are associated to the impingement of the aortic jet on the aortic wall downstream to the Valsalva sinuses, whereas in the LVOT they are due to the hypertrophic septum deviating the blood flow, consistently with the obstruction observed above in terms of velocity and pressure distribution. This is confirmed also by the line plot of Figure 10, where the WSS is shown to reach its maximum in correspondence of the maximum pressure gradient: within the LVOT region for Patients 1 and 3, at the aortic annulus for Patient 2. An interesting behavior is shown also by Patient 1: the WSS values remain non-negligible on the septal part of the aortic annulus throughout the whole systole. Since such stresses are associated to velocity gradients, this is consistent with the persistent vortical structures that can be observed in Figure 7, in the same region.

## Discussion

We introduced and applied an image-based computational approach for the analysis of the hemodynamics in patients suffering from hypertrophic cardiomyopathy. To reconstruct the geometry and the motion of the LV, we started from cine-MRI data, the gold standard for the clinical assessment of the heart function (Maceira et al., 2006; Karamitsos et al., 2009; Gulsin et al., 2017), especially regarding the diagnosis of HCM (Rickers et al., 2005; To et al., 2011; Maron, 2012). In particular, we employed standard cardiac cine-MRI data routinely acquired in diagnostic procedures, without *ad hoc* acquisition series, in order for our procedure to be applicable in virtually any clinical setting. From such data, we could generate a time-dependent, volumetric artificial image with a relatively high resolution in all directions, used to reconstruct the patient-specific geometry and motion of the left ventricle and ascending aorta during a complete heartbeat. The resulting time-dependent displacement was employed as a boundary condition for the computational fluid dynamics description of the blood flow, by Navier-Stokes equations in the ALE form. A resistive method was adopted to immerse the mitral valve leaflets in the domain.

The results of the reconstruction procedure displayed a considerable variability of the ventricle geometry and contractility among three different HCM patients. Different portions of the endocardium can be affected by hypokinesis, leading to different evolutions of the chamber volume and shape. The associated inhomogeneities in the ventricular displacement, as well as the assessment of the end-diastolic and end-systolic volume, showed how the ellipsoid approximation commonly employed in clinical evaluations may be quite inaccurate and yield a non-negligible underestimation of the ejection fraction.

In addition to the morphological data that can be extracted from clinical images, CFD simulations provide additional information on the blood flow in terms of velocity, vortical structures, and distribution of pressure and stress. To account for the domain motion, we adopted the approach of prescribed-motion CFD, which differs from the FSI approach in which the displacement of the cardiac muscle is the outcome of a mechanics model. By imposing directly the patient-specific image-based motion reconstructed from cardiac cine-MRI, we eliminated the need of a patient-dependent calibration of the myocardium mechanical model and the additional computational cost of solving the structural equations coupled with hemodynamics. This came at the expense of a more complex reconstruction procedure, described and discussed above. To the best of our knowledge, the present study represents the first comparison of the hemodynamics of HCM patients based on computational hemodynamics with image-based patient-specific endocardial displacement.

The hemodynamics results quantified the flow obstruction induced by the hypertrophy of the myocardium. For all the patients, the obstruction lasted for 30% of the duration of systole. On the other hand, the typical systolic jet through the aortic orifice had significantly higher intensity in the case of obstruction than in the other cases, and this is strongly related with the pressure gradient developed in the LVOT and in the Valsalva sinuses.

The distribution of pressure on a line along the septum allowed to quantify the intraventricular pressure gradient and to identify its position and extension. The main pressure gradient develops in a very short length, in the order of 10–15 mm, and its location is different among the patients: for Patient 2 the main obstacle to the flow is represented by the aortic annulus, whereas for Patients 1 and 3 the obstruction is within the LVOT. Therefore, for Patients 1 and 3 we can state that the obstruction is directly correlated with the HCM-induced thickening of the basal portion of the septum. Moreover, in Patient 3 a pressure drop concentrates in the subaortic portion of the LVOT: this generates a Venturi effect often acknowledged as the main cause of the SAM of the mitral valve, further obstructing the flow through the LVOT and increasing the intraventricular pressure gradient, as previously reported and discussed (Fumagalli et al., 2020).

From this assessment, we can also derive clinical indications useful in the design of the possible surgical treatment by septal myectomy. Although this procedure is well-established and typically entails low mortality and an impressive long-term survival rate (Ommen et al., 2005; Maron and Nishimura, 2014), it is currently designed combining the imaging data and the surgeon’s experience, and more detailed quantitative information may be useful. In this regard, our identification of the regions of high pressure gradient along the septum may be directly exploited during the surgical procedure, since the distances on the line that we draw, starting from the right coronary ostium and running toward the ventricular apex, can be measured on the surgical field.

Our analysis highlighted also other regions of the patients’ anatomy that should be subject to follow-up examinations, although they are not directly involved by the hypertrophy. From the evaluation of the WSS, we could measure the impingement of the systolic jet on the aortic wall, particularly in the case of Patient 2 and 3, in whom follow-up future monitoring is advisable to reduce the risk of wall damage and possible aneurysm formation (Dolan et al., 2013).

### Study Limitations

First, the present study analyzed a small number of subjects, and none of them underwent surgery so far, based on the indications given by their attending clinicians. Therefore, our study represents a proof of concept, and for the moment *ex post* clinical validation is still missing. However, being this a computational study, and not a statistical one, we designed an *a priori* model, supported by the imaging data and based on the physical principles of fluid dynamics, and not an *a posteriori* model based only on clinical measurements. As a consequence, our sample is in fact not limited, and it allowed us to provide useful quantitative information about the patients, with the possibility of future utilization for other HCM patients. For sure, investigations involving a larger number of patients will achieve a more comprehensive understanding of the hemodynamics of HCM, confirming our preliminary results, and better accounting for the strong inter-patient variability of this clinical condition. To facilitate this process, an improved automatization of the reconstruction procedure is under development, to reduce the time elapsing between the acquisition of data and the post-processing of computational results.

Second, the aortic valve leaflets are not considered in the current settings, since, in the absence of pathological features, they are expected to have a negligible influence on the development of the intraventricular obstruction, which is the main feature of interest to classify HOCM. However, including also that valve in the geometry would allow an accurate assessment of the flow in the ascending aorta.

Another limitation consists in the study of the systolic phase solely. To extend the assessment of the effects of HCM over the whole cardiac function, the model can be extended from the systolic phase to the full heartbeat (and possibly multiple heartbeats). This would entail to consider the valves opening and closing dynamics and to introduce a proper reconstruction of the left atrium: since such data is only partially available in standard cardiac cine-MRI data, an extension of the reconstruction algorithm should be envisaged.

Finally, alternative management options for HOCM, such as medical treatment with a combination of beta-blockers, calcium channel blockers, and/or antiarrhythmic medications, or other interventional approaches like septal ablation and potential implantation of cardioverter defibrillator, have not been discussed, as all of them are beyond the purpose of our computational study.

## Conclusion

Regarding our patient population:

• The hypertrophy in Patient 1 does not induce an obstruction since little intraventricular pressure gradient is appreciable. Nevertheless, the inhomogeneous displacement partially hinders the effectiveness of the cardiac pump, with an ejection fraction that is just above the limit value of 50%.
• About Patient 2, the pressure gradient mainly develops at the aortic annulus, thus it is not directly associated to HCM. Moreover, it is of small magnitude, and it does not induce a pathologically intense aortic jet. However, the relatively large LV volume and rather generalized hypokinesis determine a low ejection fraction.
• Patient 3 fits different criterions for the definition of HOCM, with a strong aortic jet accompanied by a significant pressure gradient in the LVOT, and we could also quantify the Venturi effect that may have been at the origin of the development of a SAM of the mitral valve. Moreover, the obstruction has been localized, providing relevant indications for the design of a possible surgical treatment by septal myectomy.

These conclusions confirm the suitability and effectiveness of our proposed computational approach to assess the cardiac function and the hemodynamical implications of HCM, as well as to provide clinically relevant indications potentially useful to guide the surgical treatment of the disease.

## Data Availability Statement

The raw data supporting the conclusions of this article will be made available by the authors, without undue reservation.

## Ethics Statement

The studies involving human participants were reviewed and approved by the Ethics Committee of L. Sacco Hospital. The patients/participants provided their written informed consent to participate in this study.

## Author Contributions

IF: conception of the study, development of the methods and software, image processing, simulations, analysis of the results, manuscript drafting, figures preparation, and revision of manuscript. PV: image processing, simulations, analysis of the results, figures preparation, and revision of manuscript. CV: conception of the study, analysis of the results, manuscript drafting, revision of manuscript, and supervision of the study. MF: development of the methods and software, and revision of manuscript. AC: clinical relevance of the results, and revision of manuscript. SI: images acquisition. RS: clinical relevance of the results, and revision of manuscript. AQ: funding, coordination of the study, and revision of manuscript. All authors contributed to the article and approved the submitted version.

## Conflict of Interest

The authors declare that the research was conducted in the absence of any commercial or financial relationships that could be construed as a potential conflict of interest.

## Publisher’s Note

All claims expressed in this article are solely those of the authors and do not necessarily represent those of their affiliated organizations, or those of the publisher, the editors and the reviewers. Any product that may be evaluated in this article, or claim that may be made by its manufacturer, is not guaranteed or endorsed by the publisher.

## Footnotes

1
www.mitk.org

2
simpleitk.org

3
www.vtk.org

4
www.vmtk.org

5
lifex.gitlab.io

6
www.dealii.org

7 iHEART – An Integrated Heart model for the simulation of the cardiac function. European Research Council (ERC) grant agreement No. 740132, P.I. Prof A. Quarteroni.
